# Interleaved multivoxel ^31^P MR spectroscopy

**DOI:** 10.1002/mrm.26172

**Published:** 2016-02-23

**Authors:** Fabian Niess, Georg B. Fiedler, Albrecht I. Schmid, Sigrun Goluch, Roberta Kriegl, Michael Wolzt, Ewald Moser, Martin Meyerspeer

**Affiliations:** ^1^ Center for Medical Physics and Biomedical EngineeringMedical University of Vienna, ViennaAustria; ^2^ MR Center of Excellence, Medical University of ViennaViennaAustria; ^3^ Faculty of PhysicsTechnical University of ViennaViennaAustria; ^4^ Division of Endocrinology and Metabolism, Department of Internal Medicine IIIMedical University of ViennaViennaAustria; ^5^ Department of Clinical PharmacologyMedical University of ViennaViennaAustria

**Keywords:** spectroscopic localization, dynamic ^31^P MRS, exercising muscle, multivoxel

## Abstract

**Purpose:**

Separate measurements are required when investigating multiple exercising muscles with singlevoxel‐localized dynamic ^31^P‐MRS. With multivoxel spectroscopy, ^31^P‐MRS time‐series spectra are acquired from multiple independent regions during one exercise‐recovery experiment with the same time resolution as for singlevoxel measurements.

**Methods:**

Multiple independently selected volumes were localized using temporally interleaved semi‐LASER excitations at 7T. Signal loss caused by mutual saturation from shared excitation or refocusing slices was quantified at partial and full overlap, and potential contamination was investigated in phantom measurements. During an exercise‐recovery experiment both gastrocnemius medialis and soleus of two healthy volunteers were measured using multivoxel acquisitions with a total *T*_R_ of 6 s, while avoiding overlap of excitation slices.

**Results:**

Signal reduction by shared adiabatic refocusing slices selected 1 s after the preceding voxel was between 10% (full overlap) and 20% (half overlap), in a phantom measurement. In vivo data were acquired from both muscles within the same exercise experiment, with 13–18% signal reduction. Spectra show phosphocreatine, inorganic phosphate, adenosine‐triposphate, phosphomonoesters, and phosphodiesters.

**Conclusion:**

Signal decrease was relatively low compared to the 2‐fold increase in information. The approach could help to improve the understanding in metabolic research and is applicable to other organs and nuclei. Magn Reson Med 77:921–927, 2017. © 2016 The Authors Magnetic Resonance in Medicine published by Wiley Periodicals, Inc. on behalf of International Society for Magnetic Resonance in Medicine. This is an open access article under the terms of the Creative Commons Attribution License, which permits use, distribution and reproduction in any medium, provided the original work is properly cited.

## Introduction

^31^P‐MRS has become a trusted method to analyze metabolite concentrations 1 and kinetics, e.g., in muscle tissue 2, 3. The introduction of ultra‐high field (7T and above) whole body MR scanners 4 allows the application of advanced localization techniques 5 to increase the specificity and, in combination with multichannel coil arrays, achieve sufficient signal to noise ratio (SNR) 6. Additionally, dynamic ^31^P‐MRS benefits from increased *T*_1_ relaxivity of phosphocreatine at 7T 7, which helps increase SNR per unit time and/or temporal resolution via shorter repetition times.

So far, different methods for localizing the ^31^P MR signal from exercising muscles have been presented in the literature, ranging from explicitly using the limited field of view of small surface coils over slab localization 8, 9 and single voxel spectroscopy 10, 11 to chemical shift imaging 12 and gradient echo ^31^P‐imaging 13, 14. Each of those approaches has specific benefits and shortcomings concerning SNR, time resolution, *B*_1_ profile or complexity in implementation.

The high time resolution desired for analyzing the metabolic response of muscle tissue during exercise can be accomplished using a single‐shot acquisition scheme like, e.g., semi‐LASER (localization by adiabatic selective refocusing) single voxel spectroscopy 10, 15. Data can be acquired from gastrocnemius medialis (GM) and soleus (SOL) during plantar flexion and subsequent recovery 11 in two consecutive exercise bouts. However, performing the task twice is time consuming, not trivial to reproduce with high precision, and a preceding exercise may have an influence on physiological parameters measured during the following exercises. The acquisition of several spectra from different positions during one measurement as presented in this work could therefore help to acquire physiologically more meaningful data 11.

The idea of multivoxel acquisition was demonstrated as early as 1991 by Ernst and Hennig 16, with two independent voxel positions measured in an interleaved mode using a point resolved spectroscopy (PRESS) 17 or stimulated echo acquisition mode (STEAM) 18 sequence, with ^1^ H‐MRS in the brain at 2 T. To avoid mutual saturation due to the slice selective RF‐Pulses, tilted gradients were used, which prevents any of the orthogonal slices from being shared by the volumes of interest (VOI). Recently Boer et al. 19 developed a different approach by using a multiband RF‐pulse to simultaneously excite two nonconnected slices with ^1^H‐MRS also in the brain using PRESS, semi‐LASER and STEAM. The sensitivity profile of the receiver coils were used to unfold the data with the SENSE (sensitivity encoding) approach. Both methods have their advantages and disadvantages, e.g., when using the multiband method, both voxels have to be on the same axes and cannot be set up independently. Additionally, a multiband RF‐pulse has a longer duration due to specific absorption rate (SAR) limitations, and, consequently a lower bandwidth, resulting in a higher chemical shift displacement artifact, especially at higher field strengths 19. With the approach of Ernst and Hennig, the voxel positions could be set up relatively independently, but while acquiring both VOIs in an interleaved mode, the mutual saturation of the volumes during localization must be prevented 16.

This work presents a method to localize two or more independent VOIs by temporal interleaving of semi‐LASER for acquiring multiple time series of spectra in one experiment at 7 T, which alleviates most of the constraints that come with previously presented multivoxel approaches. The feasibility and performance of the approach is demonstrated on time‐resolved ^31^P MR spectra from exercising muscles, but the concept is applicable to other nuclei and organs of interest.

## METHODS

To test the multivoxel acquisition scheme, phantom and in vivo measurements were performed. Two healthy subjects were measured (one male and one female), who had declared written informed consent according to the guidelines of the local ethics committee and the declarations of Helsinki. Two muscles, i.e., gastrocnemius medialis and soleus, were measured during rest exercise and recovery.

**Figure 1:**
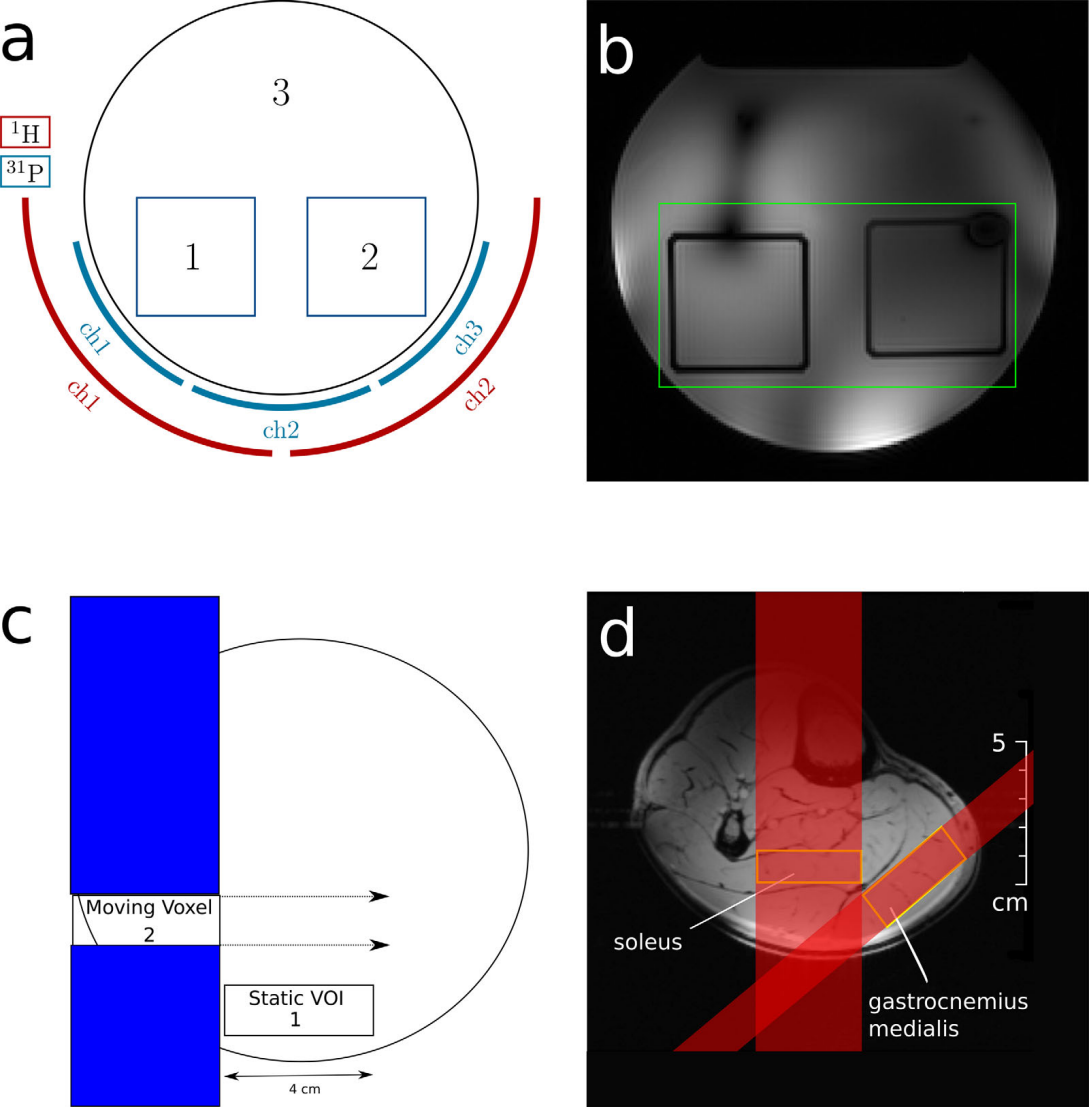
Schematic representation of the cylindrical phantom (containing two small compartments) atop the multichannel transceiver coil (**a**), with a transversal localizer image showing the compartments and the shim volume [green rectangle (**b**)]. The effect of partially overlapping voxels was quantified in an unstructured test object, by moving voxel 2 along the *x*‐axis in steps of 5 mm, while holding the position of voxel 1 constant (**c**). Transversal gradient echo image of a human calf muscle with the positions of two VOIs for acquiring ^31^P spectra from gastrocnemius medialis and soleus. Shaded sections schematically show the slice affected by the excitation pulses (**d**).

A structured phantom was built to demonstrate correct localization of multiple voxels within one scan and to investigate possible contamination of the signal by contributions from outside the selected compartment. The phantom contained two 40 mm^3^ cubic volumes placed side by side inside a 2.8 l plastic cylinder (14 cm inner diameter, see Figure 1a). A potassium hydrogen phosphate solution (50 mmol/l) was filled in both cubes and in the surrounding cylinder. The pH values of the compartments were changed using NaOH pellets to differentiate signals originating from the respective volumes, which also influenced *T*_1_ relaxation times. The signal of the left and right compartment resonated at 0.7 ppm and −0,3 ppm, with the resonance of the surrounding solution set to 0 ppm. An in‐house built three channel ^31^P, two channel ^1^H tranceiver coil, form‐fitted to the calf, was used for the measurement 6. The ^1^H channels were used for acquiring scout images and for shimming. The shim volume (green rectangle in Fig. 1b) contained both VOIs. The acquisition parameters for the phantom measurements for both volumes were set to the same values, i.e., *T*_E_ = 40 ms, excitation pulse duration *T*_exc_ = 3.5 ms and refocusing pulse duration *T*_refoc_ = 7.2 ms. The voxel orientation was chosen to ensure that all refocusing slices contained both VOIs at a full overlap. Only the excitation slices of the voxels did not overlap, to prevent unwanted mutual excitation.

Separate measurements of both VOIs with *T*_R_ = 8 s in both compartments were used as reference, and compared with results from interleaved multivoxel measurements, acquired with a total *T*_R_ of 8 s and allowing 4 s between each VOI localization. The SNR was calculated using the signal area and the noise calculated by the fitting routine of AMARES 20 using jMRUI 21. The saturation effects caused by either a shared excitation slice or (one or both) refocusing slice(s) were quantified by comparing spectra measured with the singlevoxel and multivoxel acquisition schemes, using a single‐compartment (i.e., unstructured) phantom containing 100 mmol/l potassium hydrogen phosphate at pH 5.3 (*T*_1_ = 2.91 ± 0.17 s). Measurements using a shared excitation slice or two refocusing slices were measured on different days. To pronounce the saturation effect on the right VOI, the time interval between the localization of the left and the right VOI was reduced to 1 s followed by a delay of 7 s before the next repetition (total *T*_R_ = 8 s) and vice versa. Signals were acquired with 30 repetitions; the first five scans were excluded from averaging to ensure steady‐state conditions.

Quantifying the influence of the excitation slice only was accomplished by setting the refocusing pulse voltage of the previous localization to 0 V.

In in vivo situations, it is not always possible to position voxels ensuring full overlap of all refocusing slices, therefore the influence of partially or fully overlapping refocusing slices was studied in measurements using the single compartment phantom. A $15\times 40\times 40\;{\text{mm}}^{3}$ voxel, centered along the *x* direction and close to the coil, was measured 1 s after the localization of another VOI with the same size, placed in a plane above. The measurement was repeated with the upper voxel shifted continuously in 5 mm steps along *x* (from left to right), as shown in Figure 1c. This measurement was done without and with full overlap in *z* direction, i.e., the VOIs shared one or two refocusing slices, one with full and the other with a varying partial overlap. Four spectra were averaged per position, excluding the first acquisition, to ensure steady‐state conditions.

The multivoxel MRS acquisition scheme was applied in vivo during an exercise‐recovery experiment, to simultaneously acquire data from GM and SOL. Voxels were positioned as shown in Figure 1d, avoiding overlap of excitation slices. The exercise protocol was 2 min of rest, followed by an 5 min exercise, (plantar flexion using a custom built pneumatic pedal ergometer, see 22, at 30% of maximum voluntary contraction force), and 7 min of subsequent recovery. Total *T*_R_ was 6 s, with 3 s between the excitation of each voxel. The excitation pulse duration was 2.6 ms for both VOIs, the refocusing pulse duration was 4.6 ms, *T*_E_ = 29 ms in SOL and 3.4 ms, *T*_E_ = 24 ms in GM.

Since the GM VOI was located closer to the coil than the SOL VOI, the required pulse amplitude for excitation and refocusing was lower, but as the refocusing slices of the GM VOI were overlapping with the SOL VOI, the refocusing pulse amplitude for GM was adjusted to ensure full refocusing of the SOL, with the objective to minimize saturation effects. Ten spectra were acquired in the resting muscles before the exercise‐recovery experiment using consecutive single voxel acquisitions. Equal parameters were used for the multivoxel acquisition. These single voxel data were averaged per muscle and compared with the average of the first 10 spectra from the multivoxel acquisitions. The first, fully relaxed scans were excluded.

## RESULTS

**Figure 2:**
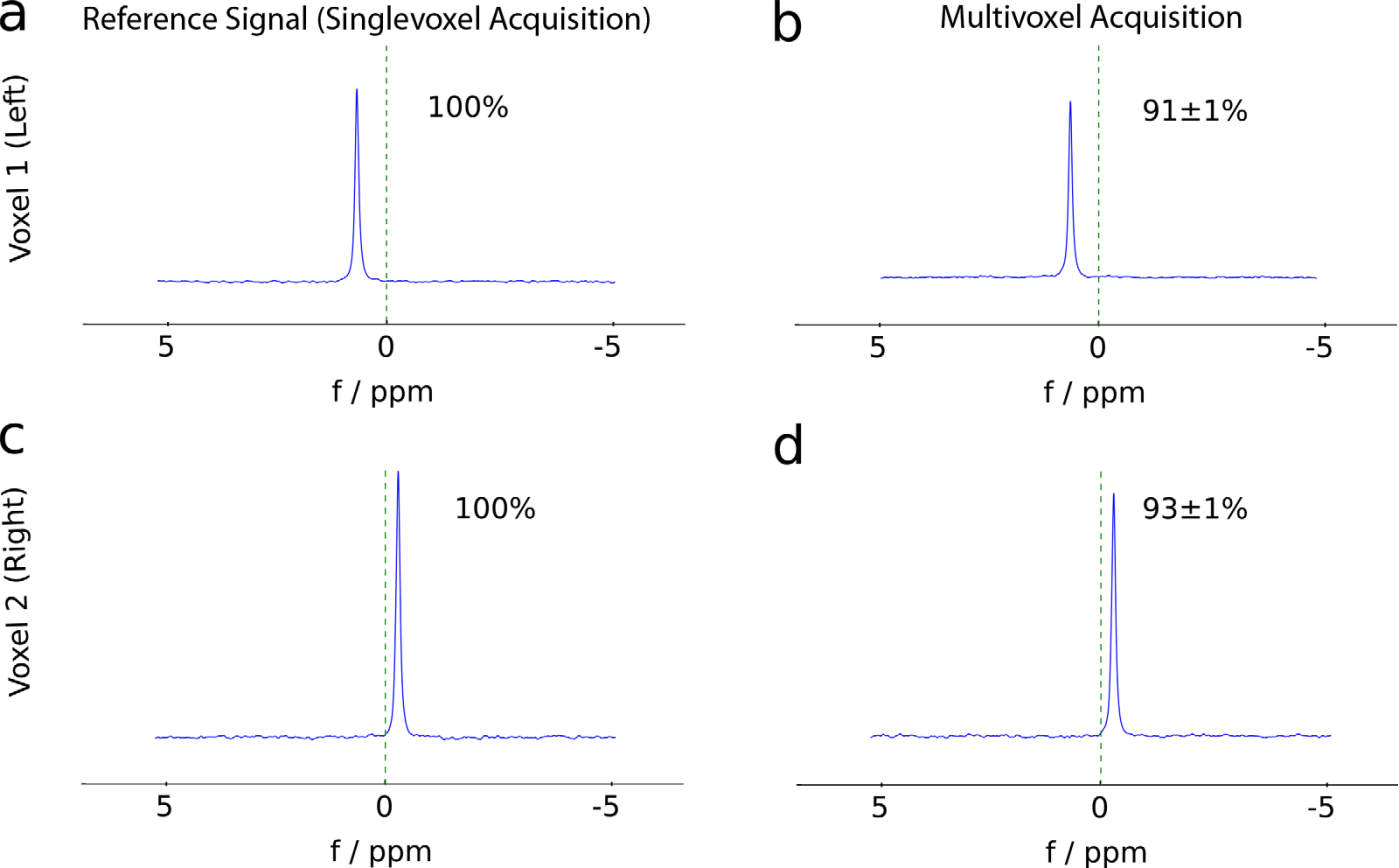
Comparison of signals acquired separately using singlevoxel spectroscopy (*T*_R_ = 8 s each) from the left (**a**) and right (**c**) compartment inside the structured phantom, with data collected with the multivoxel acquisition approach [left (**b**) and right (**d**) within *T*_R_ = 8 s with 4 s delay between each localization]. There is no visible contamination using interleaved acquisition of both VOIs. The signal loss was 9±1% in the left (b) and 7±1% in the right VOI (d). Contamination from the surrounding would result in a resonance peak at 0 ppm.

Spectra of the multivoxel acquisition of the left and right compartment in the structured phantom were similar to the reference, acquired with standard singlevoxel semi‐LASER, as shown in Figure 2. All spectra are free from any perceptible contamination by signal from the surrounding solution, which would give rise to a peak at 0 ppm. The signal loss in multivoxel acquisitions, compared to singlevoxel measurements, was 9±1% in the left and 7±1% in the right voxel, respectively.

The saturation effects caused by either sharing an excitation slice only, or by two refocusing slices fully overlapping, measured in the single‐compartment phantom, are given in Table 1. When sharing only the excitation slice, the measured signal decreased by 35–46%, 1 s after the preceding excitation of the other voxel, respectively. The signal loss incurred by the multivoxel acquisition when sharing only refocusing slices was much smaller, with 10–22% for 1 s delay and 7–10% with 4 s delay between the localizations. By increasing the refocusing pulse's voltage by 12.5%, a reduction of the signal loss was achieved, reaching 3±0.5% in voxel 1 (left) and 8±0.5% in voxel 2 (right), with a 4 s time delay between the localizations.

**Table 1:** Signals Measured from Two 35 mm^3^ VOIs Placed Side By Side Inside a Homogeneous Phantom Close to the Coil

Shared excitation slice	Area of signal peak (a.u)	SNR	Signal loss
Left voxel reference	6563 ± 49	27.2 ± 1.1	–
Right voxel reference	7693 ± 48	22.8 ± 1.1	–
Left voxel MVS shared excitation	3527 ± 12	12.5 ± 0.4	46 ± 0.3%
Right voxel MVS shared excitation	4991 ± 55	10.6 ± 0.4	35 ± 1%
Shared refocusing slices			
Left voxel reference	3994 ± 22	17.7 ± 0.6	–
Right voxel reference	6178 ± 55	17.7 ± 0.8	–
Left voxel MVS (1 + 7)	3607 ± 19	16.1 ± 0.6	10 ± 1%
Right voxel MVS (1 + 7)	4860 ± 50	14.7 ± 0.6	21 ± 1%
Left voxel MVS (4 + 4)	3737 ± 29	16.7 ± 0.8	6 ± 1%
Right voxel MVS (4 + 4)	5563 ± 56	16.4 ± 0.7	10 ± 1%
Left voxel MVS (4 + 4) + 12.5%	3880 ± 18	16.7 ± 0.7	3 ± 1%
Right voxel MVS (4 + 4) + 12.5%	5681 ± 44	16.5 ± 0.4	8 ± 1%

Both VOIs were acquired separately with *T*_R_ = 8 s as reference signals. For multivoxel spectroscopy (MVS), the delay between the localizations was 1 s, followed by a delay of 7 s with a shared excitation slice only and vice versa for each voxel position. The influence of sharing both refocusing pulses was tested with asymmetric (1 + 7 s) and symmetric (4 + 4 s) timing. To reduce saturation effects caused by refocusing pulses, the measurement was repeated with the refocusing pulse voltage increased by 12.5% (from 160 to 180 V).

**Figure 3:**
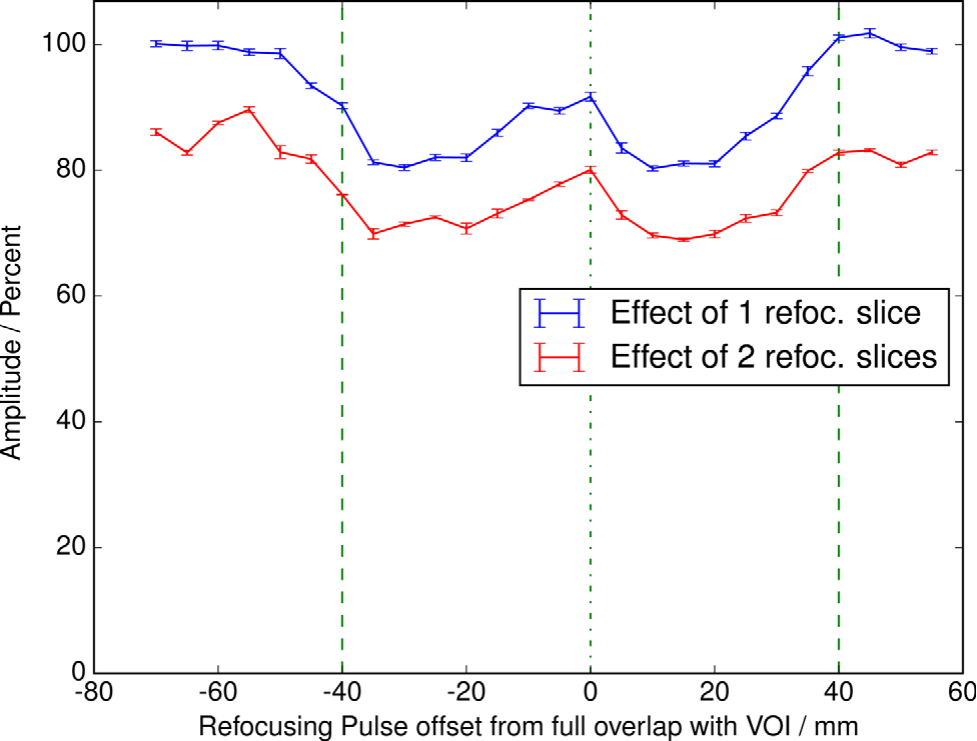
Signals of a static VOI close to the coil acquired 1 s after the two adiabatic full passage refocusing pulses were applied to localize a second voxel. The second voxel was located on the left upper corner of the measured VOI and was shifted in 5 mm steps to the right after each measurement. Blue line: influence of one refocusing slice at different overlap positions. Red line: the second refocusing slice of the moving voxel constantly fully overlaps with the VOI. Both curves show the same features, the additional refocusing pulse induces an offset by −10%. Outside the dashed lines, the nominal voxels do not overlap.

The effect of refocusing pulses for several partial overlap positions is shown in Figure 3. This is a realistic assumption for placing voxels in vivo, where it is not always possible to avoid a partial overlap, e.g. with gastrocnemius medialis and soleus muscles. To pronounce the effect, signals were measured with a 1 s delay after the excitation of the previous VOI. As shown in Figure 3, both curves show the same features, and stronger saturation was observed when the second refocusing slice was constantly overlapping (c.f., the offset of the red trace in Fig. 3). The shape of the curves implied that a full overlap caused the smallest ( 9±1%) and a partial overlap of 50% the largest signal loss ( 20±0.5%), compared to the reference for the blue curve. A second refocusing slice with full overlap in all measurements added a constant signal loss ( 10±1%), as illustrated by the red curve.

**Figure 4:**
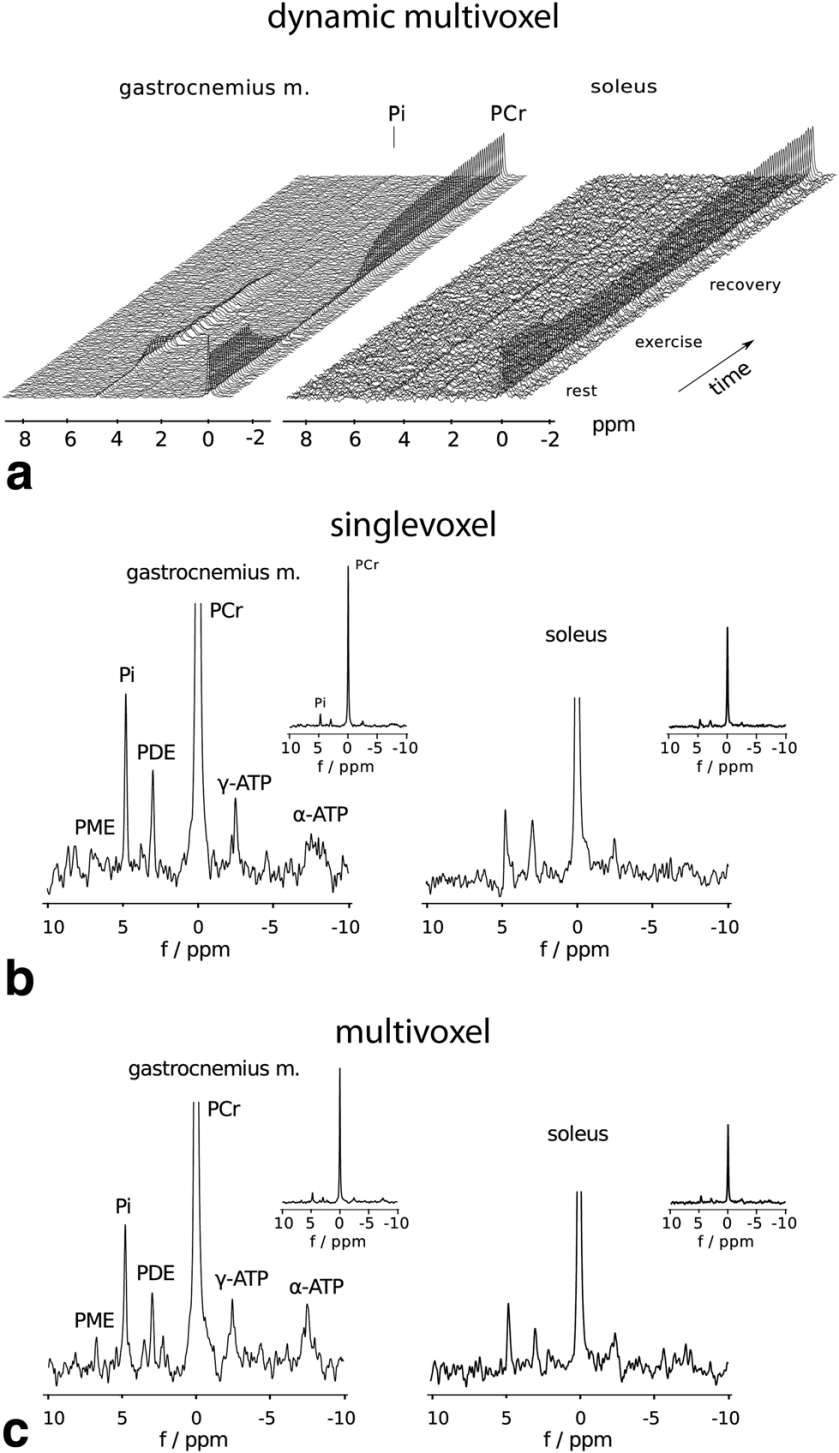
Time series of ^31^P‐MR spectra acquired in gastrocnemius medialis and soleus with a multivoxel protocol during one exercise‐recovery experiment comprising rest (2 min), exercise (5 min), and recovery (7 min). Unaveraged spectra are shown, time resolution 6 s (**a**). Spectra from gastrocnemius medialis and soleus at rest, representing 10 averaged spectra (1 min acquisition time), which were measured either separately using a singlevoxel protocol (**b**) or using the interleaved multivoxel protocol (**c**). Resting spectra have identical scale and are zoomed to metabolites, cutting off phosphocreatine, while the inserts show the full scale. All spectra were acquired with 2048 points, bandwidth of 5000 Hz and were filtered with 7 Hz apodisation and zero filled by a factor of 4 for display.

Multivoxel measurements in vivo were performed on two subjects (one male and one female), the spectra from one subject (female, age: 26 years) are shown in Figure 4. Spectral quality of the singlevoxel spectra from both muscles is comparable to the multivoxel spectra shown in Figure 4b and c. Besides phosphocreatine and inorganic phosphate, the metabolites phosphomonoesters, phosphodiesters, and *γ*‐ATP and *α*‐ATP are clearly visible. The signal loss found in vivo was lower for GM in both subjects ( 14±3% male, 13±2% female) as compared to results from SOL ( 16±4% male, 18±4% female).

## DISCUSSION

The multivoxel spectroscopy sequence produced almost the double amount of SNR per unit time compared to consecutive singlevoxel experiments, by extracting information from two relevant regions of interest in a single measurement.

Other methods to acquire similar information have their respective benefits, e.g., better coverage with ^31^P‐MRI 14, and higher specificity with ^31^P‐MRSI 23. Since a high time resolution is desired to follow the evolution of metabolite concentrations and pH during an exercise‐recovery experiment, the benefits of the multivoxel spectroscopy scheme are most pertinent to the characterization of physiological parameters of muscle tissue: semi‐LASER provides more spectral information compared to ^31^P‐MRI and is faster than ^31^P‐MRSI 14, 23, at relatively high SNR and it features a better point‐spread‐function leading to excellent suppression of outer volume contamination. In this work, we present ^31^P semi‐LASER spectra acquired simultaneously in two different muscles within 1 min, that show phosphocreatine, inorganic phosphate, phosphomonoesters, phosphodiesters, and ATP resonances (the latter with reduced SNR due to *J*‐evolution during *T*_E_). An alternative approach for multivoxel acquisition would be to interleave two image selected in vivo spectroscopy (ISIS) localizations 24, which would allow to detect the whole spectrum, including *J*‐coupled and short‐*T*_2_ metabolites with high SNR. However, for dynamic studies, a very short *T_R_* would be necessary, considering that eight scans are required for each localization, but this introduces *T*_1_ smearing 25. The reduction of *T*_1_ smearing, e.g., with an E‐ISIS scheme 26 requires even more acquisition steps, further decreasing time resolution and rendering ISIS unattractive for dynamic studies of muscle metabolism.

Both VOIs of the single compartment phantom shared two orthogonal slices which were simultaneously affected during localization by either both refocusing slices or the excitation slice only. In the latter, the saturation effects were most prominent with up to 46% signal loss. This can be easily avoided by changing the order of slice excitation and refocusing, i.e., practically by rotating the VOI accordingly. In case of shared refocusing slices only 22% signal loss was found in the worst case, with a delay of 1 s after localizing another voxel, as shown in Table 1. With a time interval of 4 s between the localizations, which can be deemed a realistic scenario as opposed to the 1 s delay chosen deliberately to pronounce saturation effects for demonstration purposes, the losses by mutual interaction of the multivoxel acquisitions were decreased to a minimum of below 10%, for both VOIs in the homogeneous phantom. The higher saturation effects in vivo compared to phantom measurements were caused by a partial overlap of at least one refocusing slice due to VOI placement in both muscles, which is consistent with data shown in Figure 3. The reasons for saturation effects caused by the refocusing pulses are presumably an imperfect refocusing within the pulses' effective refocusing bandwidth, which gets more severe with shorter repetition times, on the one hand, and their transition region, which is not negligible, on the other hand.

The transmit voltage for the adiabatic refocusing pulses had been set SAR conservatively initially, to reach adiabaticity with an tolerated 1.4% SNR deficit. To minimize signal loss due to saturation effects caused by non‐ideal refocusing at short repetition times, as used for multivoxel measurements, transmit power can be increased, to slightly compensate saturation effect losses at cost of SAR and transmit voltage that has to be tolerated by the coil. In the present case, increasing the transmit voltage by 12.5% (and almost reaching SAR limits), helped to reduce losses caused by overlapping refocusing slices from 7 to 3% and from 10 to 8%, respectively, for the two selected voxels (see Table 1). No further improvement is expected by increasing the pulse amplitude above 12.5%. Furthermore, this is often only possibly when increasing the pulse duration and hence the echo time, which in turn increases the chemical shift displacement error.

Considering that spectra are affected differently by saturation depending on *T*_R_ and mutual overlap of the VOIs, correction factors should be established by acquiring reference data from singlevoxel measurements, prior to a quantification of the metabolite concentrations from multivoxel voxel measurements. Switching between singlevoxel and multivoxel acquisition can be easily achieved.

The SOL muscle is located deeper in the calf than GM, as shown in Figure 1, which results in the requirement of a higher transmit voltage to reach the desired flip angle. The RF pulse voltages are limited by the hardware restrictions of the coil and by the respective specific absorption rate. By increasing the pulse duration, the required $B_{1}^{+}$ amplitude decreases, but the chemical shift displacement artifact also increases. For multivoxel measurements in vivo, SAR is increased further, because the refocusing slices used for localization of the GM VOI overlap with the SOL VOI, and hence, the pulse amplitude needs to be adjusted, to assure full refocusing at the position of the SOL. It was, however, possible to acquire two voxels in GM and SOL with *T*_R_ = 6 s, within SAR limits.

With a shim volume which contains both VOIs, allowing the sequence to change the voxel position after each localization, the linewidth in vivo compared to measurements from a previous study on nine subjects 11, using a smaller shim volume, is higher in SOL ( 9.0±1 Hz instead of 6.4±1 Hz) and lower in GM ( 7.7±1.3 Hz instead of 9.9±2.7 Hz). In future studies, dynamic shimming could improve both the linewidth, and the SNR. The potential benefit of dynamic shimming of multiple locations can only be exploited with an interleaved multivoxel approach as presented in this work and cannot be applied when multiple volumes are acquired truly simultaneously, as with MRI, spectroscopic imaging or a multiband MRS technique.

The approach to allow the refocusing slices to overlap with other VOIs and still be able to individually place the VOIs with few restrictions is feasible, because semi‐LASER uses a pair of adiabatic full passage pulses, which equals a 360° flip angle for each orthogonal refocusing slice. A partial overlap of one or more refocusing slices would therefore not lead to signal cancellation assuming perfect refocusing. By using a PRESS sequence with 180° refocusing pulses, the VOI arrangement would be limited to a composition that for both VOIs refocusing slices need to fully overlap with each other at perfect refocusing condition throughout the whole VOIs.

## CONCLUSION

The use of interleaved multivoxel spectroscopy is attractive because more information can be acquired within the same time as with single voxel spectroscopy. The presented method is based on semi‐LASER, it allows an easy and fast voxel placement, e.g., for disjunct VOI or even a combination of VOIs forming an irregularly shaped volume, and keeps the signal loss minimal when only refocusing slices overlap.

Applicability of the method was demonstrated by acquiring dynamic ^31^P‐MRS data from exercising muscles which has interesting potential for metabolic research because physiological parameters can be derived specifically for different muscles, involved in a single exercise. The method is not limited to a specific nucleus and can be applied in different types of experiments.
